# Pan-Cancer Analyses Reveal Oncogenic and Immunological Role of Dickkopf-1 (DKK1)

**DOI:** 10.3389/fgene.2021.757897

**Published:** 2021-11-24

**Authors:** Shuang Gao, Ye Jin, Hongmei Zhang

**Affiliations:** ^1^ College of Life Science, North China University of Science and Technology, Tangshan, China; ^2^ School of Public Health, North China University of Science and Technology, Tangshan, China; ^3^ School of Clinical Medicine, North China University of Science and Technology, Tangshan, China; ^4^ Hebei Province Key Laboratory of Occupational Health and Safety for Coal Industry, School of Public Health, North China University of Science and Technology, Tangshan, China

**Keywords:** DKK1, pan-cancer, survival analysis, immune infiltration, biomarker

## Abstract

WNT signaling pathway inhibitor Dickkopf-1 (*DKK1*) is related to cancer progression; however, its diagnostic and prognostic potential have not been investigated in a pan-cancer perspective. In this study, multiple bioinformatic analyses were conducted to evaluate therapeutic value of *DKK1* in human cancers. The Cancer Genome Atlas (TCGA) and the Genotype-Tissue Expression (GTEx) project served as data resources. The Wilcoxon rank test was performed to evaluate the expression difference of *DKK1* between cancer tissues and normal tissues. A Kaplan-Meier curve and Cox regression were used for prognosis evaluation. Single-sample gene set enrichment analysis (ssGSEA) was used to evaluate the association of DKK1 expression with the immune cell infiltration. The potential function of DKK1 was explored by STRING and clusterProfiler. We found that the expression level of *DKK*1 is significantly different in different cancer types. Importantly, we demonstrated that *DKK1* is an independent risk factor in ESCA, LUAD, MESO, and STAD. Further analysis revealed that *DKK1* had a large effect on the immune cell infiltration and markers of certain immune cells, such as Th1 and Th2 cells. PPI network analysis and further pathway enrichment analysis indicated that *DKK1* was mainly involved in the WNT signaling pathway. Our findings suggested that *DKK1* might serve as a marker of prognosis for certain cancers by affecting the WNT signaling pathway and tumor immune microenvironment.

## Introduction

Worldwide, malignant tumors have jeopardized public health (Siegel et al., 2021). WNT signaling plays a critical role in the progress of multiple cancer types (Bian et al., 2020; Peng et al., 2021; Sun et al., 2021). Aberrant WNT signaling may subvert cancer immunosurveillance (Spranger and Gajewski, 2015; Augustin et al., 2016; Hong et al., 2016). Dickkopf-1 (*DKK1*), as a WNT signaling pathway inhibitor, is involved in the development of several types of cancers (Lu et al., 2017; Zhuang et al., 2017; Igbinigie et al., 2019). *DKK1* had decreased expression in both gastric cancer (GC) and colorectal cancer (CRC), but increased expression in breast cancer (BRCA) and non-small cell lung cancer (NSCLC) (Aguilera et al., 2006; Sato et al., 2007; Li et al., 2013; Jia et al., 2016; Kasoha et al., 2018). In esophageal cancer (ESCA), *DKK1* promoted cell proliferation through the cytoskeleton-associated protein 4 (*CKAP4*)-related pathway (Shinno et al., 2018). *DKK1* was also involved in the invasion and metastasis of intrahepatic cholangiocarcinoma (ICC) cells and lymph node metastasis (Shi et al., 2013). Various studies also demonstrated the effect of *DKK1* on the prognosis of certain cancers, such as head and neck squamous carcinoma (HNSC), NSCLC, and pancreatic adenocarcinoma (PAAD) (Yamabuki et al., 2007; Han et al., 2015; Gao et al., 2018). In liver hepatocellular carcinoma (LIHC), *DKK1* could be induced by an active WNT/β-catenin signal and further contributed to patient’s poor prognosis (Yu et al., 2009). Some reports also presented that *DKK1* might serve as a target for immunotherapy (Qian et al., 2012; Betella et al., 2020). *DKK1* could affect the function of immune cells, such as T lymphocytes and bone marrow-derived suppressor cells (Katoh and Katoh, 2017). By activating CD4^+^ and CD8^+^ T lymphocytes, *DKK1* could eliminate myeloma cells in mouse models (Qian et al., 2012). *DKK1* also inhibited the secretion of IFN-γ in Th1 cells and induced the production of interleukin (IL)-4, IL-5, IL-10, and IL-13 in Th2 cells (Bais et al., 2005). The inflammation caused by tumor-specific Th1 cells could prevent cancer, but Th2 cells have the opposite function (Kennedy and Celis, 2008; Lefrancois et al., 2020). By inhibiting β-catenin to prevent clearance by natural killer (NK) cells, *DKK1* helps to sustain the stem cell-like properties of cancer cells (Malladi et al., 2016). Based on these findings, it is necessary to evaluate the role of *DKK1* in the cancer immune microenvironment.

**FIGURE 1:**
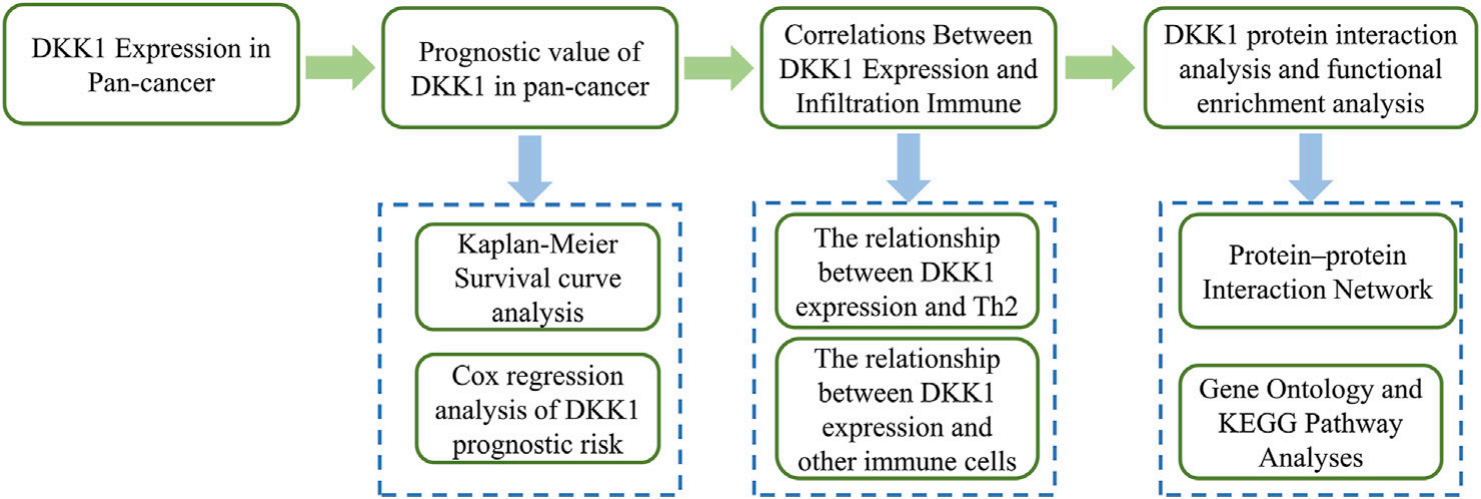
The overall flow chart of *DKK1* in pan-cancer analysis.

For several years, numerous studies have been conducted to explore the role of *DKK1* in various cancers and revealed the different roles of *DKK1* in different cancer types. In this study, we evaluated the pan-cancer expression of *DKK1* using The Cancer Genome Atlas (TCGA) dataset. Subsequently, we investigated the association of *DKK1* expression with the survival time of patients with different cancers. Finally, we analyzed the effect of *DKK1* expression on immune cell infiltration and immune cell markers. The overall process of this research is shown in Figure 1. Our findings deepened our understanding of the roles of *DKK1* in cancer progression and prognosis.

## Materials and Methods

### Pan-Cancer *DKK1* Expression Profile Analysis

The Genotype-Tissue Expression (GTEx) project and RNA-seq datasets from The Cancer Genome Atlas (TCGA) were downloaded from UCSC Xena (https://xena.ucsc.edu/) and used for pan-cancer analysis of *DKK1*. TOIL was used to reprocess the raw RNA-seq data from GTEx and TCGA databases to correct batch effects and allow for data merging across GTEx and TCGA datasets (Vivian et al., 2017). Expression differences of *DKK1* were examined using the Wilcoxon rank test with the threshold of |log_2_ FC| >1 and *p*-value < 0.05.

### Survival Analysis

Patients with different types of cancer were segregated into high and low expression groups by the median of the expression level of *DKK1*. Kaplan-Meier (KM) survival analysis was conducted by R *survival* and *survMiner* packages. Cox regression analysis was used to evaluate the relationship between *DKK1* expression and overall survival (OS) based on TCGA data. Univariate Cox analysis was performed to select relevant variables, and a multivariate Cox model was used to evaluate the independent prognostic factors. Differences were considered significant when *p*-values were less than 0.05. All analyses were carried out using R language (version 3.6.3).

### Correlations Between *DKK1* Expression and Infiltration Immune Cells

Single-sample gene set enrichment analysis (ssGSEA) was used to assess the immune cell infiltration signatures of each individual with LUAD according to the expression level of *DKK1* by using the R *GSVA* package (Hänzelmann et al., 2013). The gene set for immune cell markers was retrieved from the Laboratory of Integrative Cancer Immunology (LICI) (Bindea et al., 2013).

### Correlation Between *DKK1* Expression and Immune Cell Markers

The correlation module in TIMER ( http://timer.cistrome.org/) was used to analyze the correlation between the expression of *DKK1* and diverse immune cell markers. The list of different immune factors was obtained from the tumor immune system interaction database (Ru et al., 2019).

### PPI Network Construction and Functional Enrichment Analysis

The protein-protein interaction network (PPI network) of *DKK1* was constructed using the Search Tool for the Retrieval of Interacting Genes/Proteins (STRING, http://string-db.org) (von Mering et al., 2003) with a minimum required interaction score > 0.7. To identify the hub genes of *DKK1* PPI, the maximal clique centrality (MCC) algorithm was analyzed by the Cytohubba (Chin et al., 2014) plugin based on Cytoscape (Shannon et al., 2003). To evaluate the biological functions that *DKK1* is involved in, Gene Ontology (GO, http://geneontology.org/) enrichment and Kyoto Encyclopedia of Genes and Genomes (KEGG, http://www.kegg.jp/) pathway analyses were performed using the R package clusterProfiler (Yu et al., 2012).

## Results

### *DKK1* Expression Difference in Pan-Cancer

**FIGURE 2:**
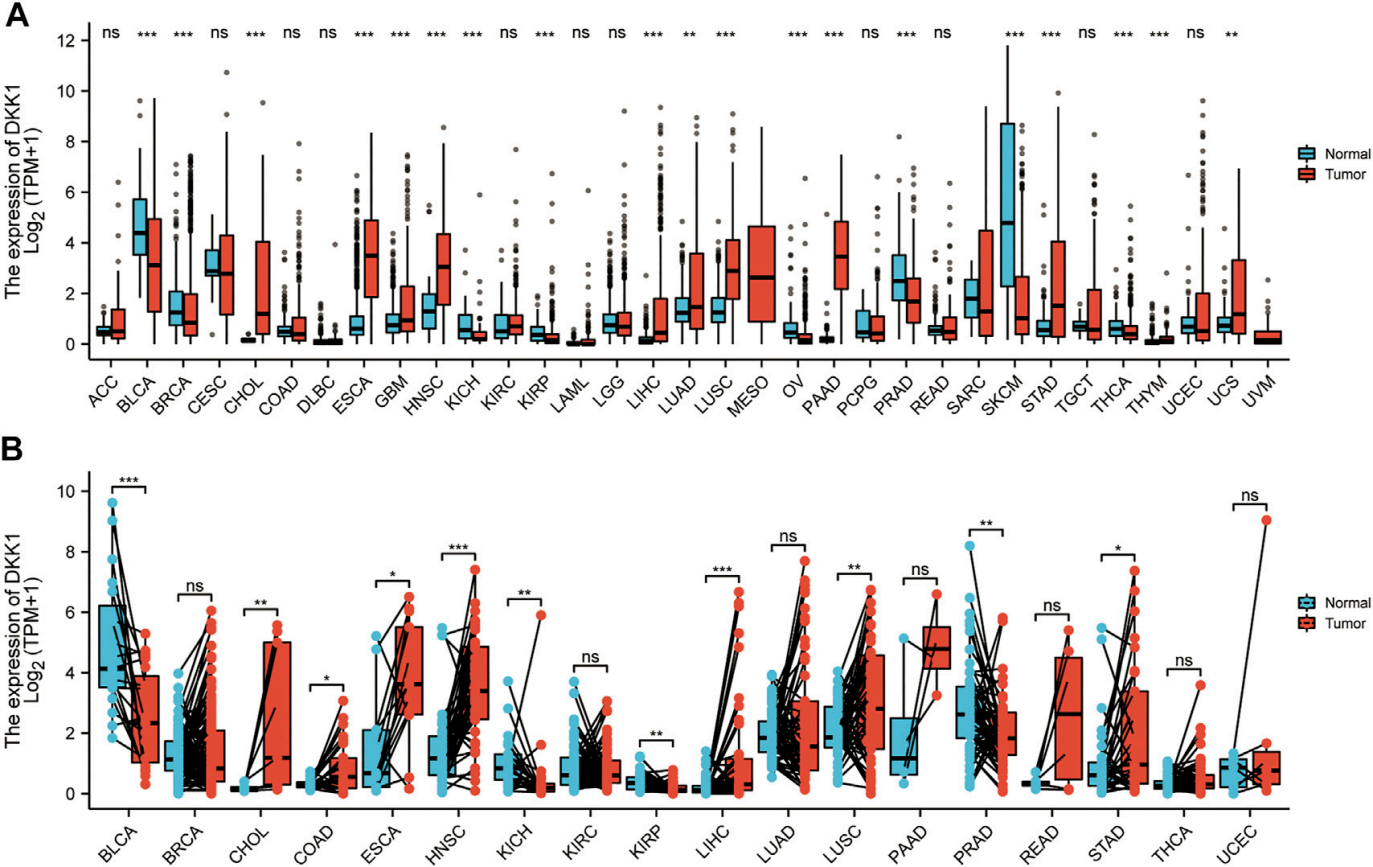
Pan-cancer analysis of differential expression of *DKK1*. The abundance is measured by log-normalized transcripts per million (TPM). **(A)** Differential expression analysis between unpaired cancer tissues and adjacent normal tissues. **(B)**. Differential expression analysis between paired cancer tissues and adjacent normal tissues.

In this study, expression difference analyses of *DKK1* were performed between cancer tissues and adjacent normal tissues. *DKK1* mRNA expression in cancer tissues from the TCGA database was inconsistent with that in GTEx and TCGA normal tissues (Figures 2A,B). *DKK1* expression was significantly higher in cancer tissues with cholangiocarcinoma (CHOL), ESCA, HNSC, LIHC, lung squamous cell carcinoma (LUSC), and STAD than that in their respective adjacent normal tissues. However, *DKK1* expression was significantly decreased in bladder urothelial carcinoma (BLCA), kidney chromophobe (KICH), kidney renal papillary cell carcinoma (KIRP), and prostate adenocarcinoma (PRAD).

### *DKK1* Expression Correlates With Cancer Prognosis and Clinical Stages

**FIGURE 3:**
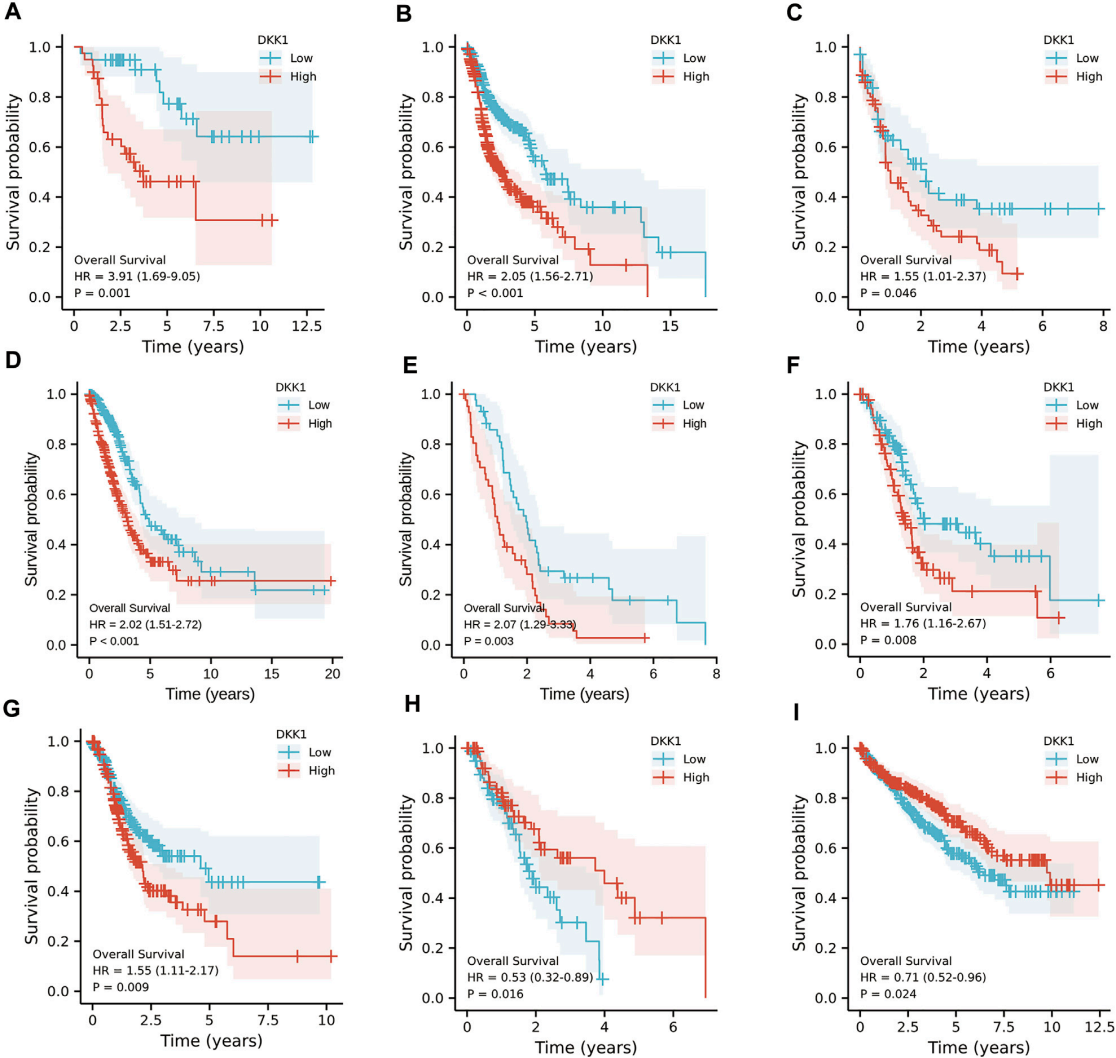
Kaplan-Meier analysis of *DKK1* expression in different cancer types. **(A–G)** Higher *DKK1* expression was correlated with the poor prognosis of ACC, HNSC, LAML, LUAD, MESO, PAAD, and STAD. **(H–I)** Lower *DKK1* expression was correlated with the poor prognosis of ESCA and KIRC.

**FIGURE 4:**
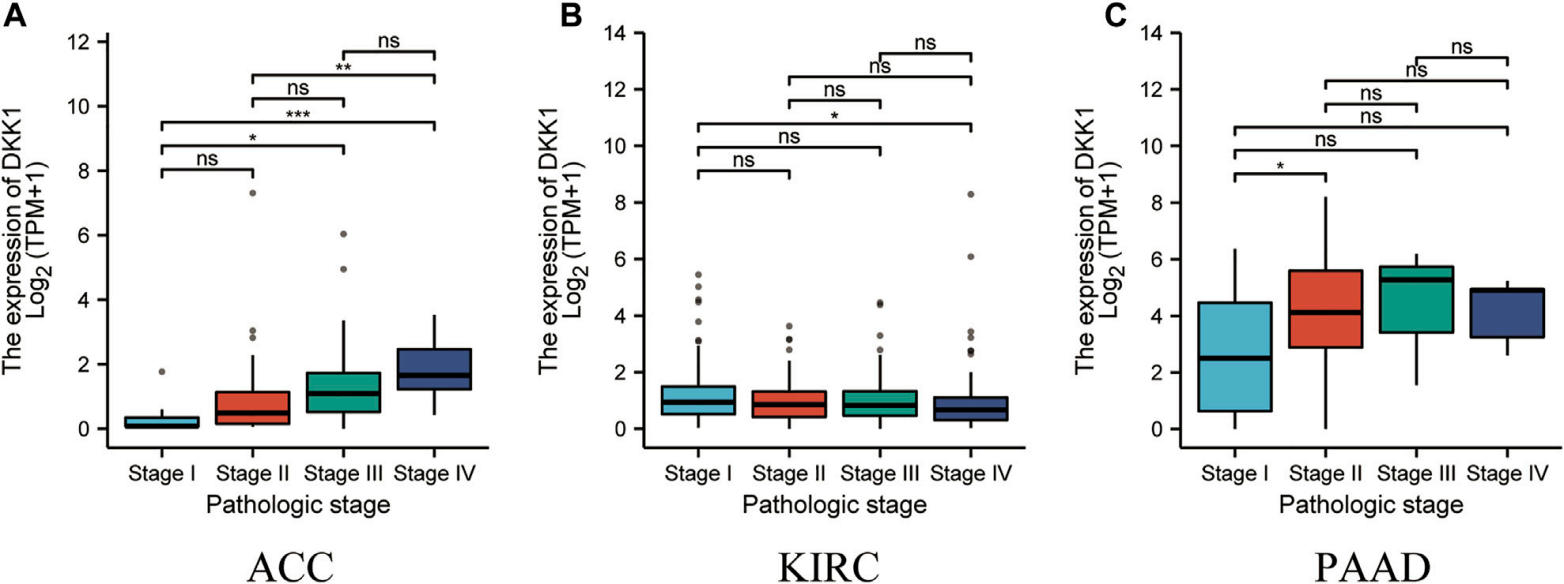
Association of *DKK1* expression with tumor stages. **(A–C)** *DKK1* expression in different stages of ACC, KIRC, and PAAD. Data shown as mean ± SD. *p < 0.05, **p < 0.01, ***p < 0.001.

To analyze the association of *DKK1* expression with clinical outcomes across all TCGA cancer types. TCGA pan-cancer analyses showed that higher *DKK1* level was significantly associated with the poor prognosis of adrenocortical carcinoma (ACC) (*p* < 0.01), HNSC (*p* < 0.01), LAML (*p* = 0.046), lung adenocarcinoma (LUAD) (*p* < 0.01), mesothelioma (MESO) (*p* < 0.01), PAAD (*p* < 0.01), and STAD (*p* < 0.01) (Figures 3A–G), and lower *DKK1* expression was significantly associated with the poor prognosis of ESCA (*p* = 0.016) and kidney renal clear cell carcinoma (KIRC) (*p* = 0.024) (Figures 3H,I). In addition, the expression of *DKK1* was closely related to the clinical stages of several cancer types, including ACC, KIRC, and PAAD (Figures 4A–C). These results indicated that *DKK1* was a potential oncogene in many types of cancer.

### DKK1 Expression as an Independent Prognostic Factor

Differential *DKK1* expression was associated with poor overall survival (OS) in several types of cancer (*p* < 0.05; Figure 3). To explore possible correlations of *DKK1* expression with clinical factors, several potential survival-related variables, including TNM stages, gender, age, smoking status, and *DKK1* expression, were entered into a multivariate Cox model. The results suggested that *DKK1* expression level was an independent protective factor for prognosis of ESCA patients [hazard ratio (HR) = 0.53, 95% confidence interval (CI) = 0.28–0.99, *p* < 0.05; Table 1), LUAD (HR = 1.95, 95% CI = 1.40–2.72, *p* < 0.01; Table 2), MESO (HR = 2.07, 95% CI = 1.29–3.33, *p* < 0.01; Table 3), and STAD (HR = 1.70, 95% CI = 1.19–2.44, *p* < 0.01; Table 4). Taken together, these results demonstrated that differential *DKK1* expression had a non-directional effect on the progression and prognosis of certain cancers.

**TABLE 1:** Univariate and multivariate Cox analyses of *DKK1* expression with overall survival (OS) among esophageal carcinoma (ESCA) patients.

Characteristics	Total (N)	Univariate analysis		Multivariate analysis	
		Hazard ratio (95% CI)	*p* Value	Hazard ratio (95% CI)	*p* Value
T stage (T3 & T4 vs. T1 & T2)	145	1.312 (0.756–2.277)	0.334		
N stage (N1 & N2 & N3 vs. N0)	144	2.970 (1.606–5.493)	<0.001	2.483 (1.221–5.049)	0.012
M stage (M1 vs. M0)	129	5.075 (2.312–11.136)	<0.001	3.378 (1.523–7.495)	0.003
Gender (male vs. female)	162	2.306 (0.922–5.770)	0.074	1.878 (0.557–6.332)	0.309
Age (>60 vs. <=60)	162	0.831 (0.506–1.365)	0.466		
Smoker (yes vs. no)	144	1.539 (0.799–2.966)	0.197		
DKK1 (high vs. low)	162	0.529 (0.315–0.888)	0.016	0.530 (0.283–0.991)	0.047

**TABLE 2:** Univariate and multivariate Cox analyses of *DKK1* expression with overall survival (OS) among lung adenocarcinoma (LUAD) patients.

Characteristics	Total (N)	Univariate analysis		Multivariate analysis	
		Hazard ratio (95% CI)	*p* Value	Hazard ratio (95% CI)	*p* Value
T stage (T2 & T3 & T4 vs. T1)	523	1.728 (1.229–2.431)	0.002	1.739 (1.117–2.709)	0.014
N stage (N1 & N2 & N3 vs. N0)	510	2.601 (1.944–3.480)	<0.001	2.524 (1.809–3.521)	<0.001
M stage (M1 vs. M0)	377	2.136 (1.248–3.653)	0.006	1.868 (1.047–3.332)	0.034
Gender (male vs. female)	526	1.070 (0.803–1.426)	0.642		
Age (>65 vs. <=65)	516	1.223 (0.916–1.635)	0.172		
Smoker (yes vs. no)	512	0.894 (0.592–1.348)	0.591		
DKK1 (high vs. low)	526	2.022 (1.505–2.717)	<0.001	1.949 (1.397–2.718)	<0.001

**TABLE 3:** Univariate and multivariate Cox analyses of *DKK1* expression with overall survival (OS) among mesothelioma (MESO) patients.

Characteristics	Total (N)	Univariate analysis		Multivariate analysis	
		Hazard ratio (95% CI)	*p* Value	Hazard ratio (95% CI)	*p* Value
T stage (T3 & T4 vs. T1 & T2)	83	0.955 (0.590–1.547)	0.852		
N stage (N1 & N2 & N3 vs. N0)	81	0.904 (0.557–1.467)	0.683		
M stage (M1 vs. M0)	59	1.917 (0.454–8.089)	0.376		
Gender (male vs. female)	85	0.944 (0.516–1.726)	0.850		
Age (>65 vs. <=65)	85	1.296 (0.805–2.085)	0.286		
DKK1 (high vs. low)	85	2.069 (1.285–3.332)	0.003	2.069 (1.285–3.332)	0.003

**TABLE 4:** Univariate and multivariate Cox analyses of *DKK1* expression with overall survival (OS) among stomach adenocarcinoma (STAD) patients.

Characteristics	Total (N)	Univariate analysis		Multivariate analysis	
		Hazard ratio (95% CI)	*p* Value	Hazard ratio (95% CI)	*p* Value
T stage (T3 & T4 vs. T1 & T2)	362	1.719 (1.131–2.612)	0.011	1.461 (0.922–2.316)	0.106
N stage (N1 & N2 & N3 vs. N0)	352	1.925 (1.264–2.931)	0.002	1.589 (1.007–2.507)	0.047
M stage (M1 vs. M0)	352	2.254 (1.295–3.924)	0.004	2.630 (1.459–4.743)	0.001
Gender (male vs. female)	370	1.267 (0.891–1.804)	0.188		
Age (>65 vs. <=65)	367	1.620 (1.154–2.276)	0.005	1.953 (1.355–2.816)	<0.001
DKK1 (high vs. low)	370	1.554 (1.114–2.167)	0.009	1.704 (1.192–2.436)	0.003

### Association Between *DKK1* Expression and Immune Responses in Cancer

**FIGURE 5:**
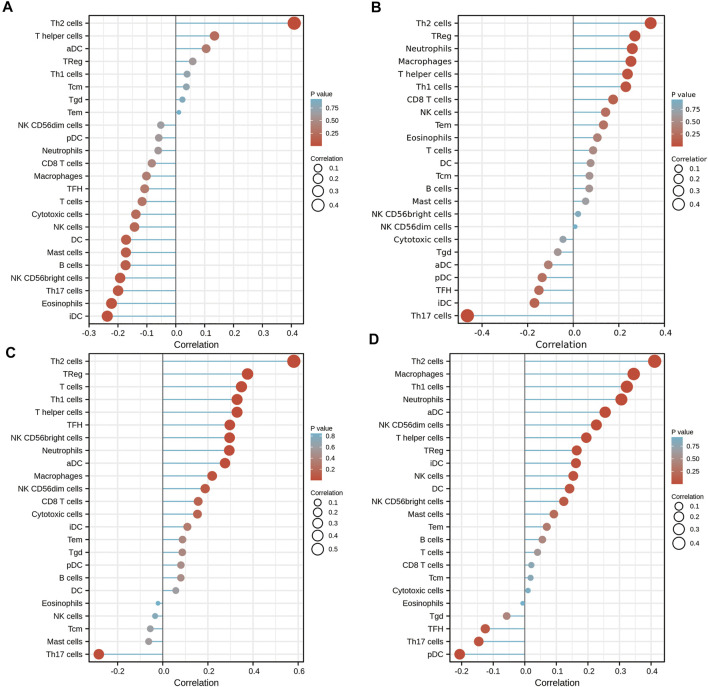
Effects of *DKK1* expression on immune cell infiltration status. **(A)** ACC; **(B)** KICH; **(C)** MESO; and **(D)** PAAD.

To further validate the role of *DKK1* as a potential immune influencer, the relationship between *DKK1* expression and immune cell infiltration was estimated. It turned out that *DKK1* was strongly correlated with the immune cell infiltration in many types of cancer (|*r*| > 0.4, *p* < 0.05). The infiltration level of Th2 cells was positively correlated with the expression of *DKK1* in ACC, KICH, MESO, and PAAD (Figures 5A–D). *DKK1* level was also correlated with immune cell infiltration of macrophages (GBM), neutrophils (PRAD), Th1 cells (SARC), NK cells (TGCT), Th1 cells (THYM), and Tgd (UVM) (Supplementary Figures S1A–F).

**FIGURE 6:**
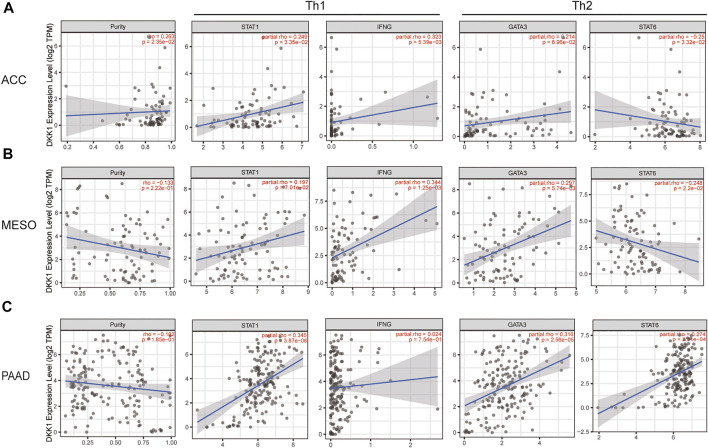
The integrated network of *DKK1*. **(A)** Protein-protein interaction network of *DKK1*; **(B)** gene-concept network for KEGG pathway analysis; and **(C)** gene-concept network for GO terms analysis.

Using the data from the TIMER database, we evaluated the correlation between *DKK1* and immune infiltrating cells. Recognizable immune cell markers included B cells, T cells (general), CD8^+^ T cells, T cells with different functions, M1 and M2 macrophages, TAMs, monocytes, NK cells, neutrophils, and dendritic cells. More directly, our findings provided evidence that the expression of *DKK1* was correlated with the level of Th1 markers (*STAT1* and *IFNG*) and Th2 markers (*GATA3* and *STAT6*) in various cancers, such as ACC, KICH, MESO, and PAAD (Figure 6; Supplementary Table S1). In addition, *DKK1* was also correlated with the level of other immune cell markers, such as GBM (macrophage markers), PRAD (neutrophils markers), and TGCT (NK cell markers) (Supplementary Table S2). These results suggested that *DKK1* might have a large impact on the tumor immune microenvironment.

### Protein–Protein Interaction Network, Gene Ontology, and KEGG Pathway Analyses

**FIGURE 7:**
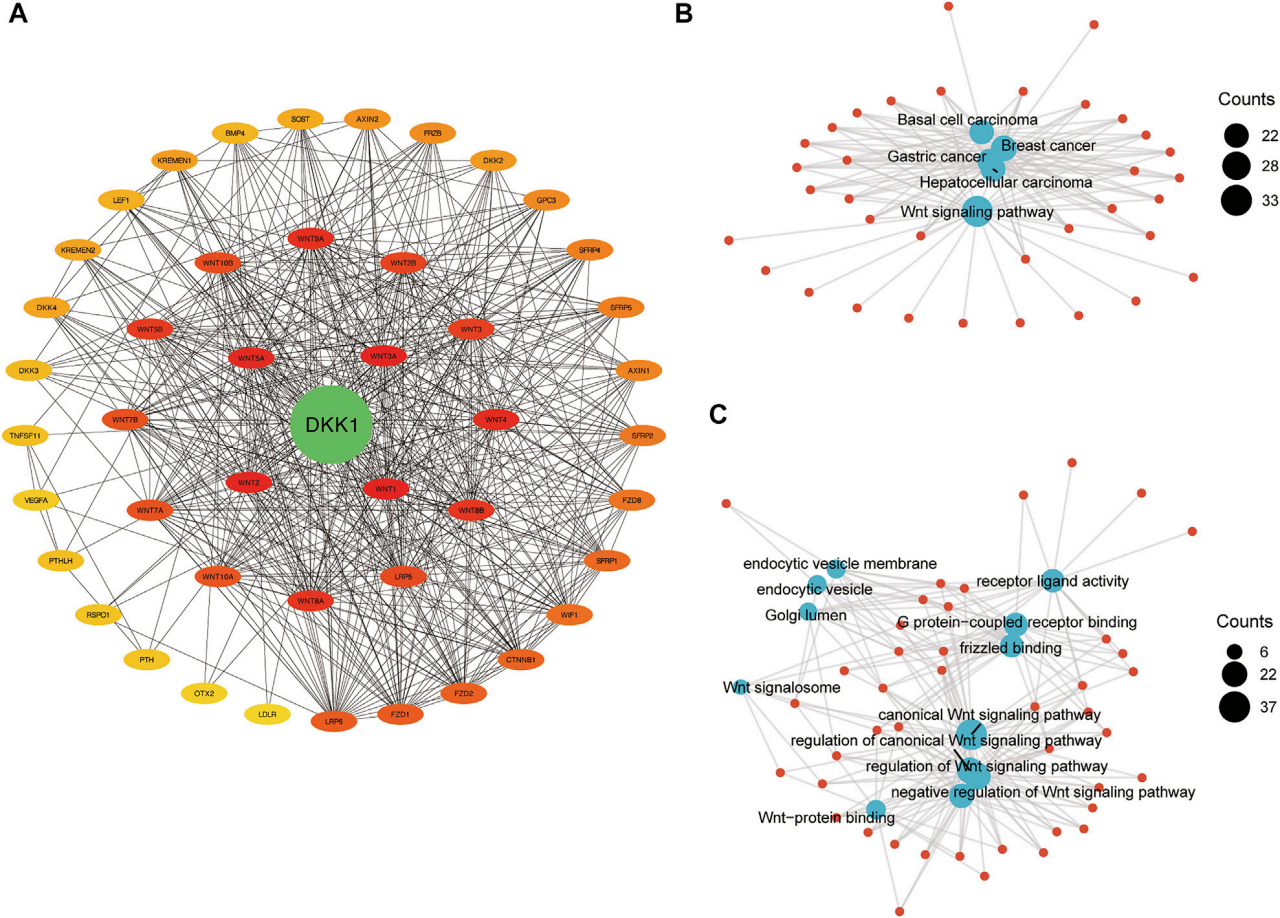
*DKK1* expression is related to the changes in Th1 and Th2 cells of ACC, LICH, MESO, and PAAD. The markers include *STAT1* and *IFNG* of Th1 cells; *GATA3* and *STAT6* of Th2. Scatter plot of *DKK1* expression in ACC **(A)** (*n* = 79), KICH **(B)** (*n* = 66), MESO **(C)** (*n* = 87), and PAAD **(D)** (*n* = 179) correlated with Th1 and Th2 cell gene markers.

A PPI network of DKK1 included 46 nodes and 506 edges. The hub genes were screened out using the Cytoscape app cytoHubba plugin. The top five hub genes were *DKK1*, *WNT1*, *WNT*2, *WNT3A,* and *WNT5A* (Figure 7A), which indicated that *DKK1* might be involved in the functional regulation of WNT family genes. To further clarify the biological functions of *DKK1*, KEGG pathway and GO terms analyses were performed. KEGG pathway analysis showed that genes in the *DKK1* PPI were mainly enriched in the WNT signaling pathway, basal cell carcinoma, breast cancer, gastric cancer, and hepatocellular carcinoma pathways (Figure 7B). GO terms were mainly concentrated in regulation of the WNT signaling pathway (biological process, BP), WNT signalosome (cellular component, CC), and frizzled binding (molecular function, MF) (Figure 7C). These results implied that genes in the *DKK1* PPI might work together to participate in cancer progression by the WNT signaling pathway.

## Discussion

*DKK1* is a well-established crucial participant in the WNT signal pathway and also acts as a major target in drug design. DKN-01, an anti-DKK1 mAb, could block the immunosuppressive effects of *DKK1* in the tumor microenvironment (TME) (Haas et al., 2021) and also perform potential antiangiogenic and immunomodulatory activity in combination therapy with gemcitabine/cisplatin in advanced biliary tract cancer (Goyal et al., 2020). Other clinical trials using anti-DKK1 mAb, such as BHQ880 (Fulciniti et al., 2009) and PF-04840082 (Betts et al., 2010) were also carried out to treat certain cancers.

The differential expression of *DKK1* has been reported in many different cancers; however, there is a lack of comprehensive pan-cancer analysis of *DKK1*. In the current study, after analyzing the expression level of *DKK1* in cancer and normal tissues of 33 cancer types, we found that *DKK1* was upregulated in CHOL, ESCA, HNSC, LIHC, LUSC, and STAD, but downregulated in BLCA, KICH, KIRP, and PRAD. Similarly, several studies demonstrated that *DKK1* was differentially expressed in a variety of cancers and affected cancer progression by changing cancer proliferation and invasion capabilities (Shi et al., 2013; Zhuang et al., 2017; Fezza et al., 2019). Our prognostic analysis showed that in most cancers (ACC, HNSC, LAML, LUAD, MESO, PAAD, and STAD), the upregulated expression of *DKK1* was associated with poor prognosis. This further supported the results from previous studies which also confirmed the relationship between the overexpression of *DKK1* and the lower overall survival in HNSC (Gao et al., 2018), NSCLC (Yamabuki et al., 2007), and PAAD (Han et al., 2015). However, in ESCA and KIRC, our data showed that a lower *DKK1* level contributed to poor prognosis. Other pan-cancer analysis also provided evidence that the same gene could result in a controversial consequence in different kinds of cancer. For example, a pan-cancer study showed that there was a significant different expression of *NLRP3* in 15 different cancers, and it was used as an independent posterior factor of SKCM (Ju et al., 2021). Similarly, when comparing the expression level of *Fam20C* in cancer tissues with that in neighboring normal tissues, we found a large variation across different kinds of cancers which indicated the different roles in different cancers (Liu et al., 2021).

The effect of *DKK1* on various cancers may be the result of abnormal activation of WNT signaling (Niida et al., 2004). Our PPI analysis and the pathway enrichment analysis showed that the genes interacting with *DKK1* were mainly involved in the WNT signaling pathway, basal cell carcinoma, breast cancer, gastric cancer, and liver cancer. *DKK*1 has been reported to inhibit the interaction of *LRP 5/6* with a *WNT* signal and the formation of the Fzd-WNT-LRP5/6 complex (Wirths et al., 2003; Ahn et al., 2011). By interfering with *DKK1*, the activated WNT3a/β-catenin signal had a great influence on cell proliferation, cell cycle acceleration, invasion, and migration (Ren et al., 2021). These results suggested that *DKK1* may participate in cancer progression through the WNT signaling pathway.

To unravel the potential mechanism of the predictive value of DKK1 alterations for the tumor immune microenvironment, types of infiltrating immune cells were surveyed. Chronic inflammation is a well-acknowledged risk factor of cancers, we hypothesized that DKK1 influenced cancer prognosis through immune cell infiltration. After conducting immune infiltration analysis, we found that the expression *DKK1* was correlated with certain immune cell markers on Th1 and Th2 cells. Wang’s study showed that Th2 cells were identified as prognostic immune cells in gastric cancer (Wang et al., 2020). Th2 cells also demonstrated therapeutic potential for adoptive cell therapy (ACT) (Lorvik et al., 2016). Recent studies demonstrated that Th2 responses played a critical role in the pathogenesis of cancers, such as luminal breast cancer (Zhang et al., 2015), prostate and advanced melanoma cancer (Dulos et al., 2012), and myeloma (Tian et al., 2019). The deep understanding of the relationship between DKK1 and Th2 responses will help us comprehend the mechanism of local antitumor response. In recent years, with the development of immune checkpoint inhibitors, infiltrating immune cell markers can not only be used as prognostic markers, but also have received extensive attention as a new type of treatment (Ladanyi, 2015).

Taken together, our study annotated *DKK1* expression in a pan-cancer manner and identified that *DKK1* could be used as an independent prognosis factor. *DKK1* was significantly expressed in various cancers, and it might also be a biomarker for tumor immunity or even targeted therapy. This study also provided evidence of the effect of *DKK1* on immune cell infiltration. However, this study has its limitations. Since all analyses were based on online datasets, experimental confirmation from a laboratory is still needed.

## Data Availability

Publicly available datasets were analyzed in this study. This data can be found here: https://xena.ucsc.edu/
