# Supplementary material for: Pan-Cancer Analyses Reveal Oncogenic and Immunological Role of Dickkopf-1 (DKK1)
Source: Front Genet. 2021 Nov 24;12:757897. doi: 10.3389/fgene.2021.757897 (PMC8654726; doi:10.3389/fgene.2021.757897)
Supplement: Supplementary file 3 [file DataSheet1.docx]

Supplementary Material


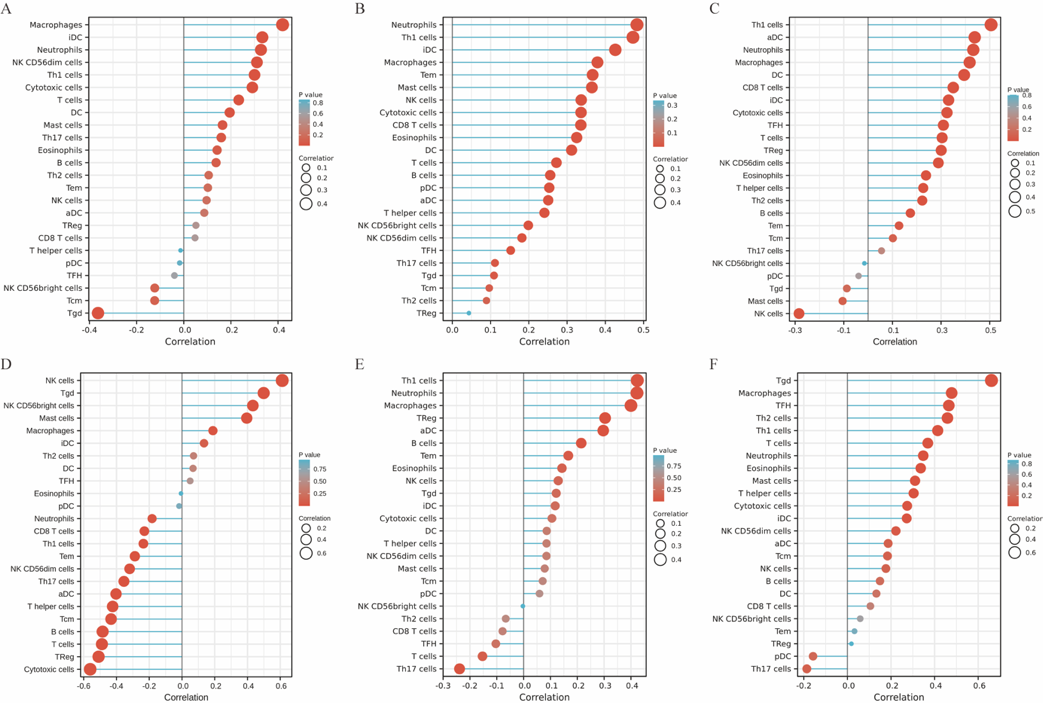


**Supplementary Figure 1.** Effects of DKK1 expression on immune cell infiltration status. (A) GBM; (B) PRAD; (C) SARC; (D) TGCT; (E) THYM; and (F) UVM**.**
